# Virtual brain models: a roadmap towards effective brain digital twins

**DOI:** 10.3389/fncom.2026.1973956

**Published:** 2026-10-29

**Authors:** Egidio D’Angelo, Claudia A. M. Gandini Wheeler-Kingshott, Alain Destexhe, Viktor Jirsa

**Affiliations:** 1 Department of Brain and Behavioral Sciences, University of Pavia, Pavia, Italy; 2 Digital Neuroscience Center, IRCCS Mondino Foundation, Pavia, Italy; 3 NMR Research Unit, Queen Square MS Centre, Department of Neuroinflammation, UCL Queen Square Institute of Neurology, Faculty of Brain Sciences, UCL (University College London), London, United Kingdom; 4 CNRS, Paris-Saclay Institute of Neuroscience (NeuroPSI), Paris-Saclay University, Gif-sur-Yvette, France; 5 Institut de Neurosciences des Systèmes, INSERM, INS, Aix Marseille University, Marseille, France

**Keywords:** digital twins, neural circuits, neurology, physiology and biophysics, virtual brains

## Abstract

Virtual Brain Models (VBMs) are emerging computational tools promising to revolutionize personalised and precision medicine. VBMs reproduce brain functional dynamics through a generative model that can be constructed and optimized against single subject data, thereby generating what is called a brain digital twin (BDT). VBMs can provide critical information about parameters of network functioning and connectivity but this ability depends on their design, raising some fundamental questions. Can VBM parameters identify key physiological features of the brain otherwise not accessible? Are VBM parameters correlated with sensorimotor and cognitive functions? What is still needed to make a VBM more precise in its physiological mechanisms? Simulations in patients affected by neurological diseases are indeed showing that VBMs can operate as effective BDTs capturing essential aspects of the physiopathology of single patients along with relevant physio-psychological correlations. However, the current VBM theory and technology still pose some limitations. We maintain that the evolution of VBM holds the key for a deeper understanding of brain physiology and pathology fostering the development of powerful computational tools capable of supporting personalized clinical decision making. Here, we will consider a roadmap addressing what VBM already do well, why this is not enough, what must change, and what becomes possible if it does.

## Foundations of virtual brain modelling

Inherent in the VBM theory is the concept of multiscale brain modelling, i.e., the ability to bridge the microscale to mesoscale and macroscale dynamic properties of the brain (D’Angelo and Gandini Wheeler-Kingshott, 2017; Einevoll et al., 2019; D’Angelo and Jirsa, 2022; Deco et al., 2008). This holds the promise to explain several fundamental physiological phenomena linking the activity of membrane molecules (e.g., ionic channels and receptors) to that of neurons and networks up to the whole brain. This is also fundamental for the definition of a brain digital twin (BDTs), i.e., *a digital copy of the brain structural organization and function reproducing brain dynamics in a single subject.* However, the full implementation of this approach is still incomplete.

In VBM, the brain is seen as a collection of active areas connected by fibre tracts (Jirsa and Haken, 1996; Jirsa and Haken, 1997). Therefore, a VBM is the result of three operations: connectome reconstruction, node functional description, and parameter tuning through optimization. VBM, by leveraging brain structure and function, can generate large-scale network dynamics (Arbib et al., 1997). It would therefore be desirable that VBMs are as close as possible to the biology of the brain, making them raffinate research tools capable of identifying the underpinnings of physiological functions and pathologies (Wang et al., 2024).

## Connectome reconstruction and node description

VBM starts with a regional reconstruction of the brain through a subdivision in areas connected by fiber bundles forming the structural connectome. This reconstruction is based on magnetic resonance imaging (MRI) including advanced diffusion-weighted (DW) and functional blood oxygenation level dependent (BOLD) sequences. The nodes are identified based on anatomical or functional parcellation of brain’s grey matter regions and contain models generating local neural dynamics, e.g., neural mass models, mean field models, or spiking neural networks (Figure 1A). What makes VBM functional are therefore local node dynamics that propagate into other nodes through the connectome generating large-scale oscillations mimicking EEG and fMRI recordings. Importantly, since the connectome can be extracted from MRI tractography in single subjects, the VBM can be personalized, setting the basis for the generation of a BDT. VBM uses a global coupling parameter, *G,* that represents the connectivity strength between nodes. This parameter is unique for any specific network under consideration or the whole brain, while the relative weight of inter-node connections is reported by the corresponding tract density *d* provided by the structural connectivity matrix. The nodes, in turn, contain parameters describing local network properties. For example, the Wong-Wang model (Wong and Wang, 2006) includes parameters of physiological relevance, the excitatory coupling (J_NMDA_), inhibitory coupling (J_GABA_), and recurrent excitation (J_rec_). These parameters are related to neurotransmission mechanisms involving NMDA and GABA receptors so that the model yields information about the excitation/inhibition (E/I) balance E/I = (J_NMDA_ + J_rec_)/(J_GABA_) (Li et al., 2025; Deco et al., 2014; Fousek et al., 2022).

**Figure 1:**
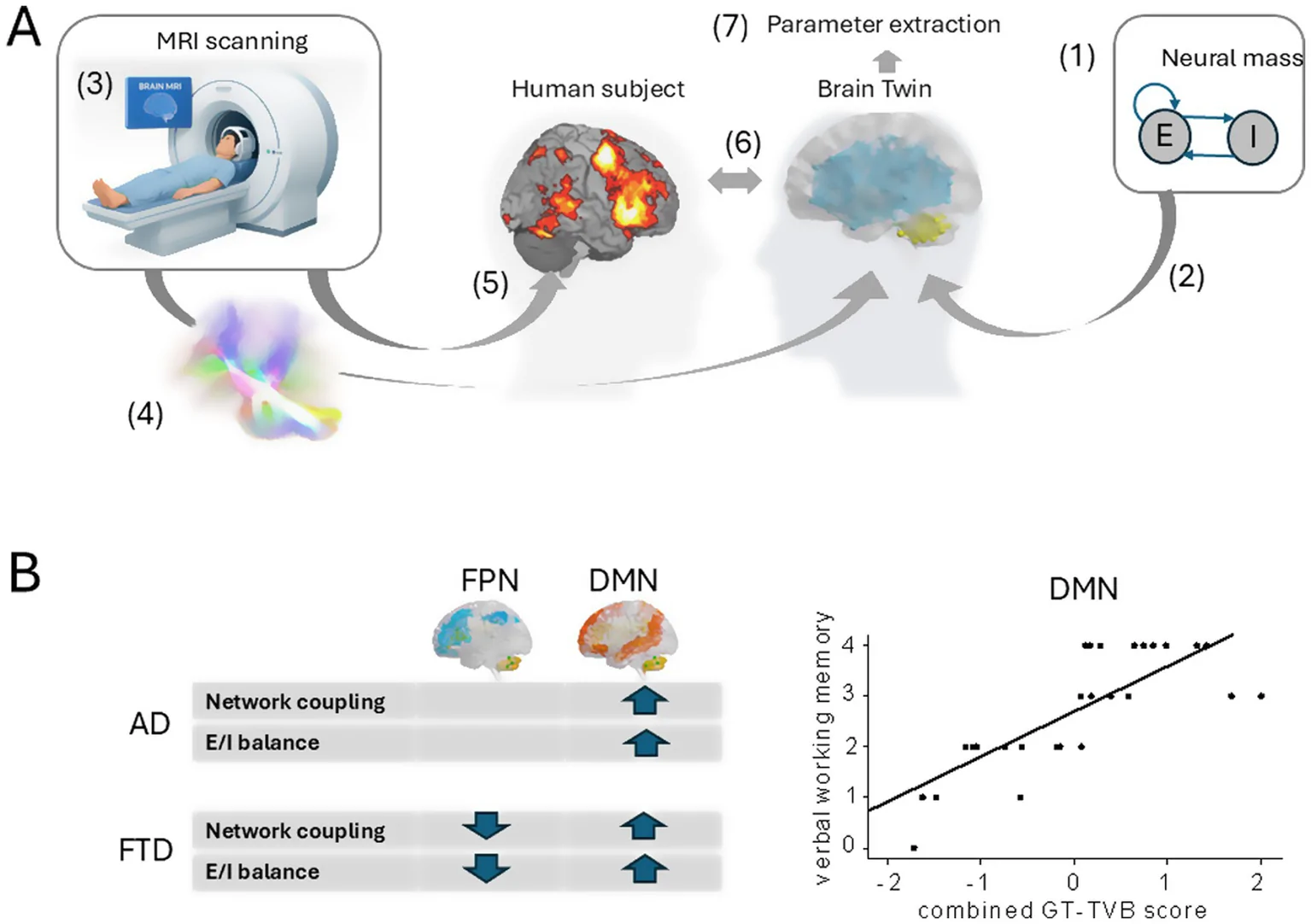
State-of-the-art brain digital twins explain the physiopathology of neurological patients. **(A)** The process of brain twin generation and simulation involves several steps. (1) Neural masses provide a generic representation of the interaction between excitatory and inhibitory neurons in a local circuit taking inspiration from the cerebral cortical column (D’Angelo and Jirsa, 2022; Spiegler and Jirsa, 2013). (2) Neural masses are placed in the nodes of the virtual brain. (3) High-field MRI scans are used to extract the brain data needed to construct the virtual brain, i.e., (4) the tractographic connectome and (5) the resting-state fMRI (Palesi et al., 2020). Finally, (6) the twin is simulated to optimize its internal parameters (7) against experimental fMRI recordings. **(B)** BDTs have been used to model the brain of patients of the dementia spectrum. The pattern of parameter changes differs considerably from AD to FTD patients, revealing DMN hyperconnectivity in both categories but selective prefrontal disinhibition only in FTD (Monteverdi et al., 2022; Monteverdi et al., 2023). In a study on MCI patients, virtual brain parameters have been correlated with neuropsychological scores taken from the same patients. There is a strong (*R*^2^ > 0.7) and significant (*p* < 0.01) correlation between circuit parameters and cognitive performance in multiple domains (in the example, verbal working memory is assessed against combined TVB and GT parameters in the DMN, which is core to learning and memory processing). These results are consistent with clinical and neurobiological knowledge about these pathologies and support the reliability of digital twin predictions about brain functioning (Monteverdi et al., 2026).

## Parameter tuning through optimization and parameter extraction

The VBM must be optimized to the individual brain through a second level of personalization instantiating model inversion through the recovery of generative-model parameters from macroscale measurements (Roy and Jirsa, 2013; Jirsa et al., 2002; Spiegler and Jirsa, 2013). Because the same macroscopic activity can be produced by many distinct microscopic configurations, the inverse problem is fundamentally degenerated (Edelman and Gally, 2001; Tononi et al., 1999), and the solution must remain at the mesoscopic scale (Lorenzi et al., 2025; Zerlaut et al., 2017; Sacha et al., 2025). Inversion is performed against data features such as static and dynamic functional connectivity (FC and DCD) and regional power spectra.

Two methodological families address the inversion problem. The first implies optimization as a reduction of the discrepancy between simulated and empirical features. A popular optimisation metric is the Pearson correlation (PCC) of the FC matrix combined with the Kolmogorov–Smirnov distance (KSD) over the FCD histogram, leading to minimize the cost function, cost = min(PCC + (1 − KSD)) (Deco et al., 2017; Deco et al., 2011; Demirtaş et al., 2016). This metric is minimized by gradient descent or evolutionary algorithms (Li et al., 2025), yielding a single best-fitting parameter vector. The second approach is probabilistic and computes the posterior distribution of plausible configurations consistent with the data, and not just a single parameter vector (Friston et al., 2019; Zeidman et al., 2023; Hashemi et al., 2025; Hashemi et al., 2023; Hashemi et al., 2021; Hashemi et al., 2020). At present, PCC-KSD optimization is the most used method to estimate the set of VBM parameter values informing about the properties of one subject’s brain (Wong and Wang, 2006).

## Identifying physiopathological features of patient’s brain with virtual brain models

A key step for VBM validation is to demonstrate its ability to capture salient alterations in patient’s brains (Figure 1B).

In pathologies of the dementia spectrum - including Alzheimer disease (AD) and frontotemporal dementia (FTD) - VBM revealed a pattern of changes matching what is known from physiopathology and anatomopathology: G and E/I balance increased in the default-mode network (DMN) of both AD and FTD, but decreased in the fronto-parietal network (FPN) of FTD (Monteverdi et al., 2022; Monteverdi et al., 2023; Monteverdi et al., 2026a). J_NMDA_ reduction in the DMN of AD was compatible with memory loss, while J_GABA_ reduction in the FPN of FTD was compatible with prefrontal disinhibition. Moreover, in mild cognitive impairment (MCI, a prodromic phase of dementia), VBM parameters showed significant correlation with neuropsychological scores over multiple cognitive domains (Monteverdi et al., 2022; Monteverdi et al., 2023; Monteverdi et al., 2026a; Zimmermann et al., 2018), so that E/I balance alterations were quantitatively correlated with learning and memory abilities on a single subject basis (Li et al., 2025). VBM parameters, by linking to the underlying molecular pathways, allowed to augment machine-learning-based classification of dementia (Stefanovski et al., 2019; Triebkorn et al., 2022).

In Parkinson’s disease, VBM integrates MRI with EEG, deep-brain electrode recordings, and structural–functional connectivity to simulate how dopamine levels shape wholebrain -dynamics, allowing to infer patient’s dopaminergic state before and after L-DOPA administration (Angiolelli et al., 2025) and to predict the impact of deep brain stimulation (Meier et al., 2022).

In cerebellar ataxias, VBM parameters reliably distinguished congenital from slowly progressive forms, revealing different combinations of alteration and compensation in motor and cognitive networks that correlated with motor and cognitive performance and explained individual clinical profiles (Gaviraghi et al., 2026).

In multiple sclerosis, VBM integrating structural connectivity and electrophysiological data have been used to estimate conduction delays, which have emerged as stronger predictors of disability than traditional markers of structural damage (Sorrentino et al., 2024; Mazzara et al., 2025; Cen et al., 2023). Moreover, J_NMDA_ revealed a strong correlation with behaviour captured by the reaction time in a sensorimotor task (Korkmaz et al., 2025).

In epilepsy, VBM integrating functional EEG and stereo-EEG data simulated whole-brain seizure dynamics improving the localisation of the epileptogenic zone (Wang et al., 2025). These VBMs reproduce how seizures start and spread using the Epileptor neural mass model (El Houssaini et al., 2020), enabling clinicians to test different stimulation strategies *in silico* before applying them to the patient (Missey et al., 2026; Jirsa et al., 2023).

This set of examples is revealing the ability of VBM to address the main categories of brain dysfunctional mechanisms: neurodegeneration (in dementia, Parkinson disease, ataxias), neuroinflammation (multiple sclerosis), and hyperexcitability (epilepsies). VBM can deal with alterations in the major brain circuits, including the thalamocortical system, cerebellum, hippocampus, and basal ganglia, that eventually control sensorimotor and cognitive-affective behaviour (D’Angelo, 2019). VBM can distinguish primary damage from compensatory reorganization and can address different physio-pathogenetic mechanisms, including genetic and epigenetic alterations, axonal conduction, synaptic transmission, and neuromodulation. Thus, alterations in high-level brain dynamics can be captured by a model based on structural connectivity and intrinsic oscillators (Wang et al., 2024) and VBM parameter can be integrated into machine-learning classification algorithms to generate BDTs improving diagnosis and simulating treatment decisions (Jirsa et al., 2023).

## Model assumptions: beyond current limitations

The surprising ability of VBMs in capturing network properties in pathologies is a proof of the efficiency of the constructive design. However, to advance VBMs beyond the current state of the art, we must consider a set of underlying assumptions about nodes and their connections that resemble symmetry, homogeneity, and isotropy in physics (Mainzer, 2013). While these assumptions hold on the large scale, they bring in some consequences and limitations that need to be relaxed to make VBMs closer to brain anatomy and physiology and of broader clinical applicability. As a drawback, increasing biological mimetics, unavoidably expands the parameter space bringing about theoretical and technical issues that are considered below.

### Symmetry of brain connectivity

The MRI connectome is taken as a proxy of brain connectivity, but there are some caveats: (1) MRI tractography and brain segmentation generate fibre tracts and nodes that do not consider neurons and axons but only bundles and masses of excitable elements; (2) MRI tracts do not contain information about the versus and sign of signal transmission; (3) MRI tracts may contain false negative and false positives; (4) Short-range connections between closely spaced regions (e.g., inside the basal ganglia circuit, cortical or cerebellar circuits) are under resolved. *De facto*, the structural connectivity matrix of the network *N* derived from MRI tractography, *M^d^ = d(N)*, is intrinsically symmetric and positive, while real brain connectivity is not. Connectivity should be rather seen as a product of matrices including sign and directionality, *M^dir^* x *M*^sign^ (Figure 2B: x is Hadamard matrix multiplication).

**Figure 2:**
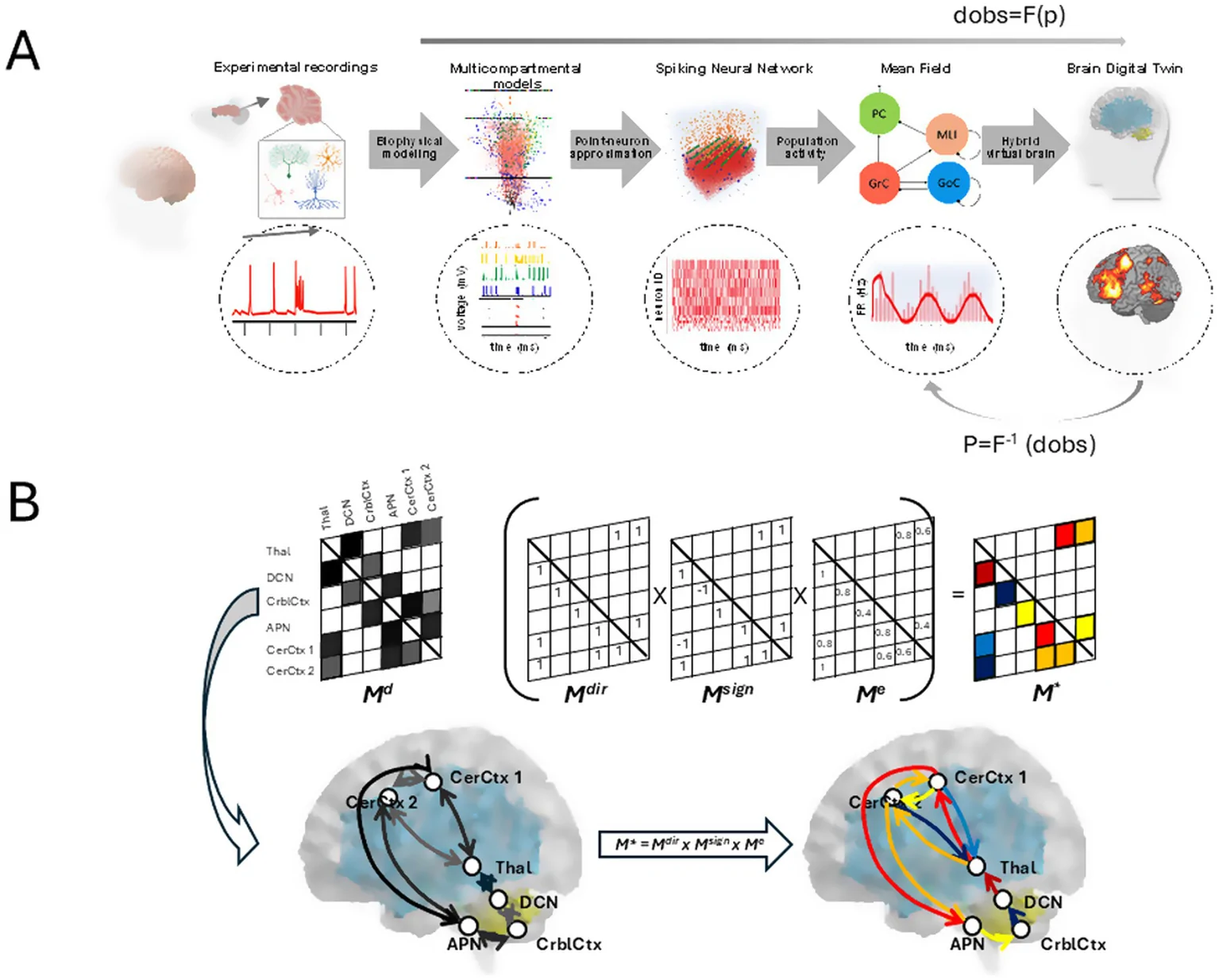
Towards more effective brain digital twins. **(A)** The homogeneity of nodes imposed by standard neural mass models can now be surpassed by MFMs specific to different neural circuits. Physiological recordings of cellular and microcircuit activity are more commonly performed in rodents, but recently, data has become available also from surgical human tissue (Masoli et al., 2024). The example shown in the figure is taken from studies on the cerebellar network. Detailed neuron models of granule cells (red), Golgi cells (blue), Purkinje cells (green), stellate cells (yellow) and basket cells (orange) were reconstructed from confocal images and patch-clamp recordings (Masoli et al., 2020a; Masoli et al., 2017; Rizza et al., 2021; Masoli et al., 2020b; Masoli et al., 2025). These models are then combined to generate modules of the cerebellar network including 10^4^–10^5^ neurons (De Schepper et al., 2022). These models can be simplified, transformed into spiking neural networks (Geminiani et al., 2024), and compressed into mean field models (Lorenzi et al., 2025), which are finally embedded into VBM nodes. This process generates ensemble signals from elementary components (D’Angelo and Gandini Wheeler-Kingshott, 2017) and, mathematically, resolves a direct problem of the type d_obs_ = F(p), in which d_obs_ is the observed signal and *p* are model parameters. The way back resolves an inverse problem of the type *p* = F^−1^(d_obs_), which can be solved by approximation (through an optimization process) on the mesoscale, i.e., giving back values for free parameters in the mean field model (D’Angelo and Jirsa, 2022). Figure panels show schematics of the models with the corresponding signals underneath. A cerebellar granule cell recording is simulated with a multicompartmental model, simplified as a point neuron, and embedded into a spiking neural network (the raster plot shows the discharge of several simulated granule cells). The PSTH shows the local field potential generated by the mean field model. Embedding into VBM allows to generate the corresponding BOLD signal in a simulated fMRI response. **(B)** The connectome extracted from MRI reconstructions is informative about the brain functional connectivity in resting-state. However, it implies a bidirectional and always excitatory node-to-node communication. The weights are determined by tract density. Now VBM evolution can leverage connectome curation. Information about the directionality of tracts and the sign of signal communication can be obtained *a priori* from literature and brain atlases. The efficiency of internode connectivity can be estimated through multiparametric optimization. The figure exemplifies the case of the cerebello-thalamo- cortical loop including the cerebellar cortex (CrblCtx), deep cerebellar nuclei (DCN), thalamus (Thal), cerebral cortex (CerCtx1, CerCtx2), and anterior pontine nuclei (APN) (Palesi et al., 2020). Anatomo-physiological evidence indicates that some tracts are unidirectional (APN→CrblCtx→DCN→Thal) and that some connections are inhibitory (CrblCtx→DCN, CerCtx→Thal). Moreover, the weights may not be the same among network nodes.

### Homogeneity of nodes

All nodes are currently represented using the same type of computational model, e.g., Wong-Wang (Wong and Wang, 2006), Jansen-Rit (Jansen and Rit, 1995), or Epileptor (El Houssaini et al., 2020). But this homogeneity is not the case in nature, since local microcircuits are the most diverse. Moreover, node representations are oversimplified, since neural masses usually consist of just two elements, one excitatory and one inhibitory, that are reciprocally and recurrently connected, summing up the properties of a multitude of different neuron types along with their specific synaptic interactions. The neural mass representation captures essential dynamical properties common to most brain circuits, including the E/I balance, recurrent excitation, and oscillations. However, they miss the diversity and algorithmic capacity and the distributed parallelized processing of local microcircuits (see Figure 2A).

### Globality of parameters

In the optimization process, parameters are often considered global rather than local variables. While this restricts the parameter space and improves the computational efficiency during model optimization, it prevents the independent tuning that would be needed to generate a physiologically meaningful circuit. The most important parameters to consider are the inter-node communication (network coupling), communication delays, and the intra-node synaptic strength.

In VBMs, *G* represents global coupling for the whole brain or for a specific brain circuit and the relative weight of connections is reported by the tract density, *d,* provided by the structural connectivity matrix. However, local connections between nodes should be set independently keeping into consideration the synaptic efficacy, *e*, i.e., the ability of a given tract to transmit (which is relatively independent from its size). Thus, in the network *N*, communication between two nodes, A→B, now depends on *d*(A→B) but this matrix should be substituted by *e*(A→B). By extending this process to all *N* nodes yields the matrix *M^e^ = e(N)*. It should be noted that *d* is known, since it is extracted from MRI tractography, while *e* is a free model parameter. In principle, *M^d^* could be used for model initialization but should eventually be substituted by *M^e^*, which could be extracted through model optimization (see below and Figure 2B).

In VBMs, tract conduction velocities ***v*** have a constant value and internodal distances ***s*** is often measured as the Euclidean distance between nodes (rather than the actual tract length), yielding the matrix of transmission delays *Δt* = *s*/*v*. However, delays should be recalculated from *a priori* independent estimates of *v* and *s*, as they critically impact on node synchrony and therefore on the simulated functional connectivity matrix (Sorrentino et al., 2024).

In VBMs, local node parameters extracted by optimization are usually kept the same in all the nodes of a specific network or of the whole brain. But this is not the case for the natural brain. Mathematically, the problem changes from a unique parameter set *P*(*N*) = *P_k_*(*N*) to the parameter matrix *P*(*N*) = *P_i,j_*(*n_i_*), where *n_i_*€*N* are network nodes, *P_i,j_* are the *j* parameters of node *i,* with *j > k*. Clearly, *P_i,j_*(*n_i_*) > > *P_k_*(*N*).

## What is the solution?

Breaking the symmetry of the network, the homogeneity of nodes, and the globality of parameters (Fousek et al., 2022; Sacha et al., 2025) implies to consider the three equations:

1. Internode communication: M* = M^dir^ x *M*^sign^ *x M*^e^.
2. Internode delay: *Δt* = *s*/*v*.
3. Node parameters: *P*(*N*) = *P_i,j_*(*n_i_*).

This approach expands the parameter space, bringing about the need of priors for *M^dir^*, *M*^sign^, and *Δt*, and of new techniques to extract parameter values through optimization for *M*^e^ and *P*(*N*).

To embed further *a priori* information into equations 1–3, graph theory and EEG metrics could be integrated into *e*(*N*) to improve inter-node communication and spectral information (Lorenzi et al., 2025; Monteverdi et al., 2026b). Further information could be obtained from dynamic causal modelling (DCM) for node communication, MRI spectroscopy for the E/I balance, near-infrared spectroscopy (NIRS) for blood flow, FDG-PET for local node metabolism, and MRI axonal diameter estimates for conduction delays (Lupi et al., 2025).

To embed biological information into the VBM nodes, specific circuit models are needed, e.g., mean field models (MFM) (Zerlaut et al., 2017; Sacha et al., 2025), which are generated bottom-up transferring biological knowledge about neurons, synapses, and molecules into the nodes (Lorenzi et al., 2025). Region-specific MFMs are available for the cerebellum, neocortex, thalamocortical circuit, basal ganglia circuit and hippocampus CA1 circuits (Tesler et al., 2024; Lorenzi et al., 2023; Goldman et al., 2020; Tesler et al., 2026; Overwiening et al., 2024). It should be noted that the cerebellum, due to its repetitive and crystallized structure, is an excellent example of how a SSN can be compressed into an effective MFM that can effectively operate within VBMs and improve their performance (Lorenzi et al., 2025; Lorenzi et al., 2023). Nonetheless, the MFM derivation procedure is agnostic with respect to network organization and, provided appropriate simplification criteria are used, it can be applied to any brain region.

To improve the connectome requires eliminating false positives and giving directionality and sign to fibre tracts. This means introducing anatomical and physiological information to create masks that can be superimposed to the MRI structural connectivity matrix (Palesi et al., 2020; Gaviraghi et al., 2025). This can be done by remapping non-human species into humans and requires careful validation. Tracer-derived projection patterns were transferred from macaque to humans using cross-species connectivity blueprints exploiting the *CoCoMac* atlas. The biological relevance of this transformation is revealed by topological analysis, which manifests the small-world network organization and directionality-based hierarchy typical of human brain networks. Moreover, directionality reshapes the propagation and persistence of sensory inputs and captures the empirical spatiotemporal dynamics of stimulus-evoked brain activity in humans. However, corrections are needed for critical connections and regions that may not be properly represented.

To accurately estimate the parameters in VBMs the traditional PCC-KSD optimization methods (Li et al., 2025) should be substituted with Bayesian frameworks like Virtual Bayesian Inference (VBI) (Ziaeemehr et al., 2025). It should be noted that increasing the number of independent network states would expand the number of free parameters leading to overparameterization. Therefore, strategies providing more constraints to the model need to be adopted to regularize parameter explosion. For example, transcriptomic gradients and gene expression data from the Allen Human Brain Atlas could provide general constraints valid for any healthy brain, while PET receptor maps, HD-EEG power spectra, and graph theory metrics could be obtained in individual subjects.

## The physiology of virtual brain models: missing mechanisms and theoretical choices

While results indicate that VBMs can provide information about latent microscopic variables by observing macroscale phenomena (D’Angelo and Jirsa, 2022), a critical analysis raises several questions. Despite the struggle of molecular and cellular physiology to define the finest details of membrane and synaptic mechanisms, large-scale brain dynamics can be captured by models based on structural connectivity and intrinsic oscillators (Deco et al., 2008; Deco et al., 2014; Deco et al., 2017; Deco et al., 2011; Cayco-Gajic et al., 2017; Schirner et al., 2023; Deco et al., 2013; Schirner et al., 2018; Deco et al., 2021; Petkoski and Jirsa, 2019; Petkoski and Jirsa, 2022). A conservative conclusion might be that, at the large-scale, the dominant effects are only partially sensitive to details at lower scales. But this explanation is unsatisfactory since, while brain organization and function are reflected into these dynamics, brain operations and computation are not (Schirner et al., 2023). So, what is missing? One possibility is that VBMs do not yet incorporate mechanisms like dendritic processing, spike timing, and synaptic plasticity. Another one is that VBMs do not have the oriented and specific connectivity subtending sensorimotor and cognitive functions and are not yet connected to sensory systems or a body, being therefore “locked-in,” without any feed-back or feed-forward system connecting them to the world. Finally, VBMs are deprived of any learning capabilities and cannot change their functional state, as real brains do. In other words, VBMs are still missing essential mechanisms and architectures that are thought to subtend brain functioning, preventing them from behaving as a biological system characterized by adaptability, intelligence, and consciousness (Koch, 2004; Llinás, 2002).

## Towards effective brain digital twins for clinical and technological applications

At this point let us speculate about a plausible future with a horizon of 5–10 years moving from the first to second generation VBMs with enhanced potential for clinical and technological translation in DBTs. VBMs incorporating mean-field models and a curated connectome (Figure 2) promise more reliable simulations of physiological and pathological mechanisms. MFM can transfer biological details and local network dynamics into the VBM, along with precise representations of pathological alterations at the molecular and cellular level (Lorenzi et al., 2025). The curated and signed connectome imports the orientation and sign of interregional communication in the VBM and can therefore identify organized communication pathways laying the basis of brain function (Jirsa and Haken, 1997; Li et al., 2025; Singer, 2009; Thivierge and Marcus, 2007). Thus, this second generation VBMs will open the way to analyze the physiology of the self-organizing nervous system in conditions like stimulus–response tasks, cognitive tasks, or learning tasks broadening the applicability to medicine and technology.

While second generation VBMs are advancing, a third wave of changes is already under development. In contrast to analog rate-coded firing rates commonly used for node activity, the impact of more realistic impulsive spike-coded neuronal discharges is being explored both in mouse (Melozzi et al., 2017; Geminiani et al., 2026) and human VBMs (Meier et al., 2022; Tartarini et al., 2026). Neuromodulator systems have been incorporated to dynamically regulate brains states (Angiolelli et al., 2025; Martin et al., 2025). Moreover, robotic controllers incorporating spiking neural networks and synaptic learning rules are converging toward the generation of embodied VBMs capable of body-environment interactions in closed-loop generating autonomous learning, sensory prediction, and adaptation (D’Angelo et al., 2025). Beyond this, the computational capacity of high-performance computing (HPC) systems also poses limits, as it is neither efficient nor fast enough to cope with real-time extended VBM simulations. Paradoxically, to effectively simulate the brain, a neuromorphic computer based on the architecture and rules of the brain might be needed to emulate the brain (Gandolfi et al., 2022; Bogdan et al., 2021)! Therefore, to move VBMs toward a mechanistic physiological interpretation, future developments will require advancements on several fronts, including physiology, physics, informatics, AI, and ultimately ethics to control the outcomes of what might become the door for advanced brain-like machines applicable in medicine and technology.

## Data Availability

The original contributions presented in the study are included in the article/supplementary material, further inquiries can be directed to the corresponding author.
